# Genetic analysis challenges the presence of *Ixodes inopinatus* in Central Europe: development of a multiplex PCR to distinguish *I. inopinatus* from* I. ricinus*

**DOI:** 10.1186/s13071-023-05971-2

**Published:** 2023-10-09

**Authors:** Kristyna Hrazdilova, Ondrej Danek, Alena Hrbatova, Barbora Cervena, Eva Noskova, Peter Adamik, Jan Votypka, Andrei Daniel Mihalca, Mechouk Noureddine, David Modry, Ludek Zurek

**Affiliations:** 1 Department of Chemistry and Biochemistry, Mendel University, Brno, Czech Republic; 2 Biomedical Center, Faculty of Medicine in Pilsen, Charles University, Plzen, Czech Republic; 3 Institute of Parasitology, Biology Center of Czech Academy of Sciences, Budějovice, Czech Republic; 4 Department of Veterinary Sciences, Faculty of Agrobiology, Food and Natural Resources/CINeZ, Czech University of Life Sciences, Prague, Czech Republic; 5 CEITEC, University of Veterinary Sciences, Brno, Czech Republic; 6 Institute of Vertebrate Biology of the Czech Academy of Sciences, Brno, Czech Republic; 7 Department of Botany and Zoology, Faculty of Science, Masaryk University, Brno, Czech Republic; 8 Department of Zoology, Palacky University Olomouc, Olomouc, Czech Republic; 9 Department of Parasitology, Charles University, Prague, Czech Republic; 10 Department of Parasitology and Parasitic Diseases, University of Agricultural Sciences and Veterinary Medicine of Cluj-Napoca, Cluj-Napoca, Romania; 11 Department of Microbiology, Nutrition and Dietetics, Faculty of Agrobiology, Food and Natural Resources/CINeZ, Czech University of Life Sciences, Prague, Czech Republic

**Keywords:** Tick, *Ixodes ricinus*, *Ixodes inopinatus*, 16S rDNA, *TROSPA*, *COI*, *ITS2*, Algeria, Czech Republic

## Abstract

**Background:**

*Ixodes ricinus* is an important vector of several pathogens, primarily in Europe. Recently, *Ixodes inopinatus* was described from Spain, Portugal, and North Africa and then reported from several European countries. In this study, a multiplex polymerase chain reaction (PCR) was developed to distinguish *I. ricinus* from *I. inopinatus* and used in the surveillance of *I. inopinatus* in Algeria (ALG) and three regions in the Czech Republic (CZ).

**Methods:**

A multiplex PCR on *TROSPA* and sequencing of several mitochondrial (16S rDNA, *COI*) and nuclear markers (*TROSPA*, *ITS2*, *calreticulin*) were used to differentiate these two species and for a subsequent phylogenetic analysis.

**Results:**

Sequencing of *TROSPA*, *COI*, and *ITS2* separated these two species into two subclades, while 16S rDNA and *calreticulin* could not distinguish *I. ricinus* from *I. inopinatus.* Interestingly, 23 nucleotide positions in the *TROSPA* gene had consistently double peaks in a subset of ticks from CZ. Cloning of these PCR products led to a clear separation of *I. ricinus* and *I. inopinatus* indicating hybridization and introgression between these two tick taxa. Based on a multiplex PCR of *TROSPA* and analysis of sequences of *TROSPA*, *COI*, and *ITS2*, the majority of ticks in CZ were *I. ricinus*, no *I. inopinatus* ticks were found, and 10 specimens showed signs of hybridization. In contrast, most ticks in ALG were *I. inopinatus*, four ticks were *I. ricinus*, and no signs of hybridization and introgression were detected.

**Conclusions:**

We developed a multiplex PCR method based on the *TROSPA* gene to differentiate *I. ricinus* and *I. inopinatus*. We demonstrate the lack of evidence for the presence of *I. inopinatus* in Central Europe and propose that previous studies be re-examined. Mitochondrial markers are not suitable for distinguishing *I. inopinatus* from *I. ricinus.* Furthermore, our data indicate that *I. inopinatus* and *I. ricinus* can hybridize, and the hybrids can survive in Europe.

**Graphical abstract:**

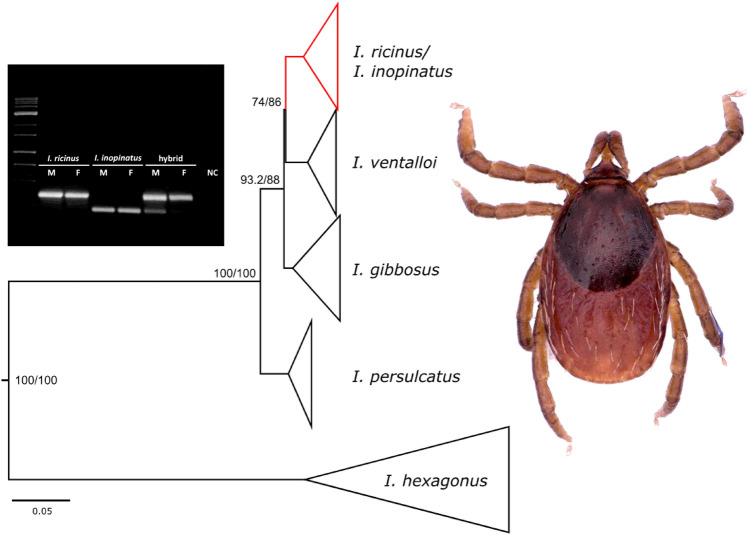

**Supplementary Information:**

The online version contains supplementary material available at 10.1186/s13071-023-05971-2.

## Background

*Ixodes ricinus* (Acari, Ixodidae) is an important vector of many pathogens of medical and veterinary importance with a wide host and distribution range across the entire West Palearctic region, from British Islands to Russian Ural and from North Africa to Scandinavia [1].

Despite the complexity of the genetic structure of *I. ricinus* populations*,* two patterns have been consistently shown, regardless of the approach used: the genetic distance of ticks on the British Islands compared with that of populations in North Africa [2–4] and the genetic dissimilarity between the North African tick population and that on continental Europe [5, 6]. In 2014, the latter pattern resulted in a description of the new species *Ixodes inopinatus*, based on the morphological characteristics and the partial sequence of the 16S rRNA gene of ticks from Spain (type locality), Portugal, and North Africa (Algeria, Tunisia, Morocco) [7].

Since then, ticks referred to as *I. inopinatus* have been reported in several European countries including Germany, Austria, Switzerland, Romania, and Turkey. These reports are based solely on morphology [8–12], morphology combined with sequencing of 16S ribosomal DNA (rDNA) [13–18], and sometimes using other genetic markers [2, 19–21]. However, with the rising number of available sequences, the number of reports of a failure to distinguish the two species has been growing, resulting in the use of the term *I. ricinus*/*inopinatus* complex [22–25]. In our surveillance of *I. inopinatus* in the Czech Republic, we also encountered difficulties in differentiating *I. ricinus* from *I. inopinatus* based on the morphology and sequencing of 16S rDNA. This resulted in our search for other genetic and easy-to-use markers that would differentiate these two sympatric and morphologically close to identical species.

So far, no consensus has been reached on the selection of a single molecular marker to differentiate ixodid tick species. The short fragment of mitochondrial 16S rRNA is often the first choice for tick identification together with the morphological description [26–28], followed by sequencing of the cytochrome *c* oxidase I subunit (*COI*) [29–31]. As for nuclear markers, the internal transcribed spacer 2 (*ITS2*) was used not only for species delineation [32–34] but also for the detection of natural hybrids between *Ixodes persulcatus* and *I. ricinus* as well as between *I. ricinus* and *I. pavlovskyi* [35, 36]. Use of other genes is scarce and in the context of *I. ricinus*/*inopinatus*, only the tick receptor for the *OspA* (*TROSPA*) and defensin genes showed discrimination power [5, 19–21].

In this study, we aimed for an easy and fast method for differentiation of the closely related and possibly sympatric species, *I. ricinus* and *I. inopinatus*, and searched for *I. inopinatus* in the Czech Republic. During the validation of a multiplex polymerase chain reaction (PCR) targeting the *TROSPA* gene, we were driven to a deeper study of the genetic diversity of these two species using mitochondrial and nuclear markers.

## Methods

Adult ticks were collected by flagging in the Czech Republic (CZ) and Algeria (ALG) between 2015 and 2020. Ticks were collected in three different regions in CZ: Libava, northern Moravia (*n* = 114), Prostredni Porici, central Moravia (*n* = 110), and Podyji, southern Moravia (*n* = 103), and from one locality in ALG: El-Tarf province (*n* = 47). Based on the morphological characteristics according to Estrada-Peña et al. [7], all ticks were identified as *I. ricinus* or *I. inopinatus*. For phylogenetic purposes, another five *Ixodes* spp. were used: *I. frontalis* (Italy, 2021), *I. gibbosus* (Italy, 2021), *I. hexagonus* (Czech Republic, 2021), *I. persulcatus* (Russia, 2019), and *I. ventalloi* (Italy, 2013). These ticks were collected by our team as part of other ongoing projects with DNA of *I. ventalloi* obtained from colleagues from Italy [37]. Ticks were identified by BLASTn (Nucleotide Basic Local Alignment Search Tool) analysis of their 16S sequences. All samples were stored in 70% EtOH at −20 °C until further analysis.

Genomic DNA was isolated from a longitudinal half of each tick using the Exgene Cell SV mini 250p Kit (GeneAll, Seoul, Korea) according to the standard protocol for animal tissues with 100 µl of elution buffer added in the final step. The other half of the tick was stored for potential reanalysis.

For an easy and fast way to distinguish the two main variants of the *TROSPA* gene sequences reported as *I. ricinus* and *I. inopinatus*, we designed a multiplex PCR. A specific pair of primers for each variant was designed within the intron region based on the alignment of available sequences in GenBank (Table 1). The resulting amplicons differed by 126 base pairs (bp) for an easy on-gel identification. PCR was performed in a total volume of 25.0 µl using 2× PCRBIO Taq Mix Red (PCR Biosystems, UK), 0.4 µM of each of the four primers and 2.0 µl of template DNA. Reaction conditions followed manufacturer instructions with the annealing temperature of 52 °C and the elongation time of 15 s for 40 cycles.

**Table 1:** Primers used in this study

Gene	Primer name	Sequence (5′—> 3′)	Product size	T_a_	References
*TROSPA*—multiplex	Trospa Iric_F1	GTAAACATCGGCCTAATGG	362 bp	52 °C	This study
	Trospa Iric_R2	GGAAAAAATAATGTTAAAACACC			
	Trospa Iino_F2	GTTGTTCACAGCGAATACT	233 bp		
	Trospa Iino_R1	GAAAAAAATATTAGAACATTAACACTC			
*TROSPA*	Trospa-F2	TATGGACACGGCGTCGCTGTC	824 bp/670 bp^b^	65 °C	Noureddine et al. [5]
	Trospa-R2	GCCCAAGCGCATAAATAAGAAGCGG			
*ITS2*	58SRSF3	CTCTTTGAACGCACATTGCGGCCT	831 bp	62 °C	Fukunaga et al. [32]
	28SRLR2^a^	TCTCGCCTGATGT**G**AGGTCGA			
*Calreticulin*	Cal_Fw1	CCCAAGGTGTACCTCAAGG	1238 bp	61.5 °C	This study
	Cal_Rev1	TCCTCTTTATCCTTCTTCTCCG			
16S rRNA	16S-F	TTAAATTGCTGTRGTATT	455 bp	52 °C	Lv et al., [55]
	16S-R1	CCGGTCTGAACTCASAWC			
*COI*	COI-F	GAATTAGGACAACCAGGA	1397 bp	55 °C	Noureddine et al. [5]
	COI-R	AAAGTATGCTCAGAAGGG			

*T*_*a*_ Annealing temperature

^a^Bold base in the primer modified from the reference

^b^Expected and amplified size of the product

For validation of the multiplex PCR and for subsequent sequence analysis, the 824 bp long fragment of the *TROSPA* gene including the entire intron was amplified and sequenced. To assess the genetic variability in more detail, fragments of two nuclear (*ITS2* and *calreticulin*) and two mitochondrial genes (16S rRNA and cytochrome C oxidase subunit I—*COI*) were also amplified and sequenced. Primer sequences and PCR conditions are shown in Table 1.

Amplification of *TROSPA*, 16S rDNA, and *ITS2* was performed in a total volume of 25 µl using 2× PCRBIO Taq Mix Red (PCR Biosystems, UK), 0.4 µM of each primer and 2 µl of template DNA. Reaction conditions followed manufacturer instructions for 40 cycles. *COI* and *calreticulin* genes were amplified in the total volume of 20 µl using Phusion Green Hot Start II High-Fidelity PCR Master Mix (Thermo Fisher Scientific, USA), 0.5 µM of each primer, and 2 µl of template DNA. Reaction conditions followed manufacturer instructions for 40 cycles. All PCR reactions were visualized on 1.5% agarose gel with the Midori Green Advance system (Nippon Genetics Europe, Germany). All products of expected length were cut from the gel, purified by the Gel/PCR DNA Fragments Extraction Kit (Geneaid Biotech Ltd., Taiwan), and sequenced by the Macrogen capillary sequencing services (Macrogen Europe, Netherlands) in both directions using the amplification primers.

Obtained sequences were assembled and visually inspected using Geneious R11.1.5 [38]. The identity of the amplicons was confirmed by BLASTn analysis (NCBI GenBank). Due to the appearance of double peaks in otherwise high-quality sequences (*TROSPA* and *calreticulin* genes), the detection of heterozygotes was performed using the Geneious plugin Find Heterozygotes followed by visual inspection and assigning ambiguous bases in positions with double peaks detected in both strands (with settings of peak similarity 30% and peak detection height 50%).

Representative samples with a high number of double peaks detected in the *TROSPA* gene as well as samples with a sudden loss of sequencing signal followed by an apparently mixed product chromatogram in the *ITS2* region were cloned using pGEM^®^-T Easy Vector Systems (Promega Corporation, USA). Acquired plasmid DNA was purified from the bacterial culture using the GenElute™ Plasmid Miniprep Kit (Sigma-Aldrich, USA) and sequenced by the Macrogen capillary sequencing services (Macrogen Europe, The Netherlands) using universal T7/SP6 primers.

For phylogenetic analyses, sequences representing 16S rDNA, *COI*, *TROSPA*, *ITS2*, and *calreticulin* from various tick species within the genus *Ixodes*, preferably from different studies and geographical origins, were selected from the GenBank. Sequences originating from this study were limited to representative sequences in the case of 16S rRNA and *COI*, representative sequences and unique clones for *ITS2*, all sequences with 0–5 ambiguities and representative clones for *TROSPA*, and all sequences with a maximum of two ambiguous nucleotides for *calreticulin* (Additional file 2: Table S2).

Phylogenetic analyses were conducted by the ClustalW alignments built in Geneious R11.1.5 [38]. After manual editing of poorly aligned regions (especially the 16S rRNA gene), phylogenies were calculated by the maximum likelihood method using IQ-TREE multicore version 2.1.3 [39]. The best-fit evolution models were chosen based on the Bayesian information criterion (BIC) computed by ModelFinder [40]. Branch support was assessed by the ultrafast bootstrap (UFBoot) approximation [41] and by the SH-like approximate likelihood ratio test (SH-aLRT) [42]. Trees were visualized and edited in FigTree v1.4.4 and Inkscape 1.1.1.

## Results

In total 374 adult ticks preselected by morphology as the *I. ricinus*/*inopinatus* complex were screened by multiplex PCR (Table 2) and three gel patterns were observed (Additional file 1: Figure S1). A single band of the size corresponding to the *I. ricinus* allele was observed in 321 ticks (317 from CZ and 4 from ALG) and a single band corresponding to the *I. inopinatus* allele was seen in 43 ticks from ALG and none from CZ. In 10 ticks from CZ, two bands were detected, each corresponding to one of the two species (Table 2, Additional file 1: Figure S1).

**Table 2:** Tick identification by multiplex PCR from four localities

Locality	*n*	*Ixodes ricinus*		Hybrids		*Ixodes inopinatus*	
		F	M	F	M	F	M
CZ Libava	114	55	55	3	1	0	0
CZ Podyji	103	45	54	2	2	0	0
CZ Prostrední Porici	110	49	59	1	1	0	0
ALG El-Tarf	47	4	0	0	0	20	23
Total	374	153	168	6	4	20	23

*n* number of ticks; *F* female; *M* male

**Fig. 1:**
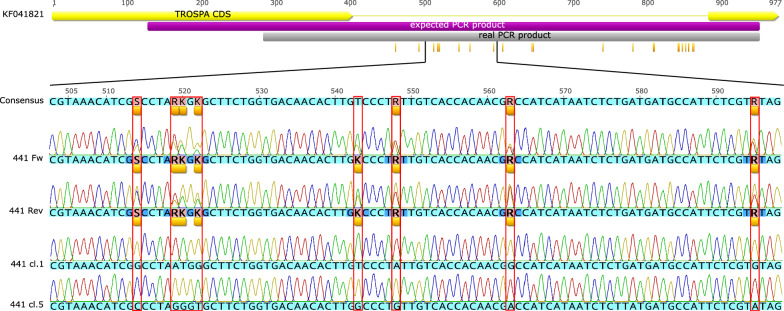
Schematic representation of the partial *TROSPA* gene sequences depicting the nucleotide positions with the double peaks (small yellow bars) (KF041821 is used as a reference sequence). The chromatograms depict the forward and reverse strands of sequencing for the uncloned PCR product. 441 cl.1 and 441 cl.5 are sequences after cloning resolving the double peaks of hybrid ticks

To validate the newly designed assay, the entire intron region of the *TROSPA* gene was amplified and sequenced from randomly selected representatives of both species from all localities and all 10 ambiguous samples. We were able to consistently amplify and sequence 670 bp out of the expected length of 824 bp, resulting in high-quality chromatograms from 112 *I. ricinus* and 19 *I. inopinatus* (based on the multiplex PCR) and all 10 ambiguous samples. Chromatograms commonly revealed double peaks; in fact, only 13 samples had no double peaks. In 117 samples, 1 to 15 clear double peaks in otherwise flawless chromatograms were observed, and in 10 samples (all assigned as ambiguous by multiplex PCR) 25 to 32 double peaks were detected in both strands (Fig. 1).

By cloning and sequencing of PCR products of the *TROSPA* gene from two ambiguous ticks and their alignment to sequences without double peaks from our study as well as with sequences from GenBank, we identified 23 positions consistently different between *I. ricinus* and *I. inopinatus* alleles (Fig. 1, Additional file 2: Table S1). In all 10 samples yielding bands corresponding to both *I. ricinus* and *I. inopinatus* in the multiplex PCR, the positions of double peaks corresponded to 23 single-nucleotide polymorphisms (SNPs) differentiating the two species. All other positions with double peaks showed no regular pattern and were detected in random positions.

**Fig. 2:**
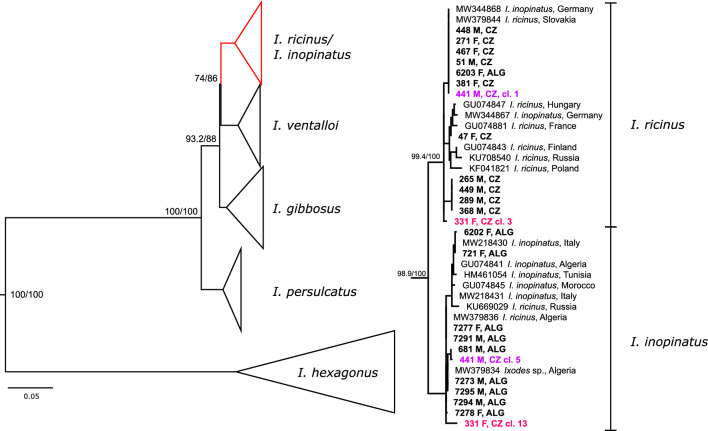
Phylogenetic tree of ticks based on the *TROSPA* gene samples from this study are indicated in bold font. *CZ* Czech Republic, A*LG* Algeria. 441 M CZ cl.1/cl.5 and 331F CZ cl.3/13 are sequences after cloning of hybrid ticks showing a clear split to *I. ricinus* and *I. inopinatus* branches

To support the above-described analyses and to put the *TROSPA* species differentiation power in a larger context, we amplified and sequenced the same fragment from other *Ixodes* spp. (minimum of three individuals per species). In the phylogenetic analyses, *I. ricinus* and *I. inopinatus* sequences from this study together with sequences from the GenBank database form two well-supported sister clades (Fig. 2). The cloned sequences of the ambiguous samples based on the multiplex PCR, the two variants of alleles representing the *I. ricinus* and *I. inopinatus* species, fell within the respective clades. All other *Ixodes* spp. form well-supported and distinguished monophyletic clades (Fig. 2, Additional file 1: Figure S2).

**Fig. 3:**
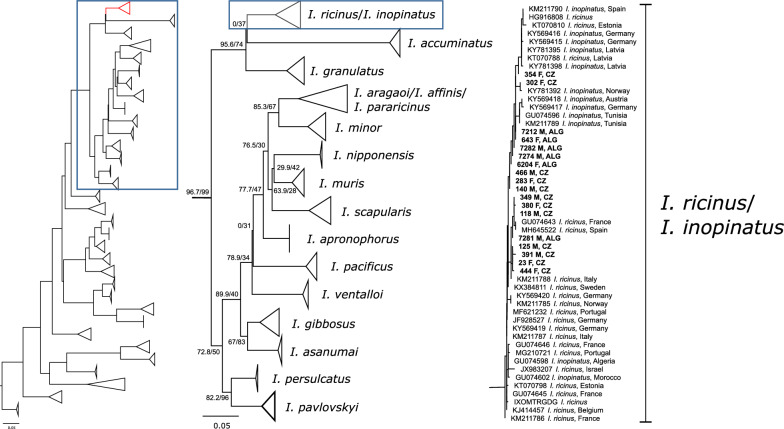
Phylogenetic tree of ticks based on the 16S rRNA gene showing the lack of power to distinguish *I. ricinus* and *I. inopinatus.* Samples from this study are indicated in bold font. *CZ* Czech Republic, *ALG* Algeria

Fragments of two mitochondrial genes, 16S rDNA and *COI*, the most commonly used molecular markers for ixodid ticks, were amplified and sequenced. The fragment of 16S rDNA was amplified and sequenced from 222 ticks from CZ and 43 ticks from ALG. Thirty-six unique haplotypes (26 CZ and 10 ALG) with sequence similarity 96.02–99.73% were identified. From these unique haplotypes, seven (three CZ and four ALG) had the “AG” haplotype assigned previously to *I. inopinatus*, 28 (23 CZ and five ALG) had the “CT” haplotype referring to *I. ricinus* [13, 16], and one new AT haplotype (1 ALG) was detected. In the phylogenetic analyses, all representative sequences from this study form a single, highly supported clade together with the *I. ricinus* and *I. inopinatus* sequences retrieved from the GenBank database. However, no structure based on the species or geography was detected within the clade(s) (Fig. 3). Other *Ixodes* spp. form well-distinguished and supported clades (with the exception of *I. affinis* and *I. pararicinus* forming a single clade, Additional file 1: Figure S3).

**Fig. 4:**
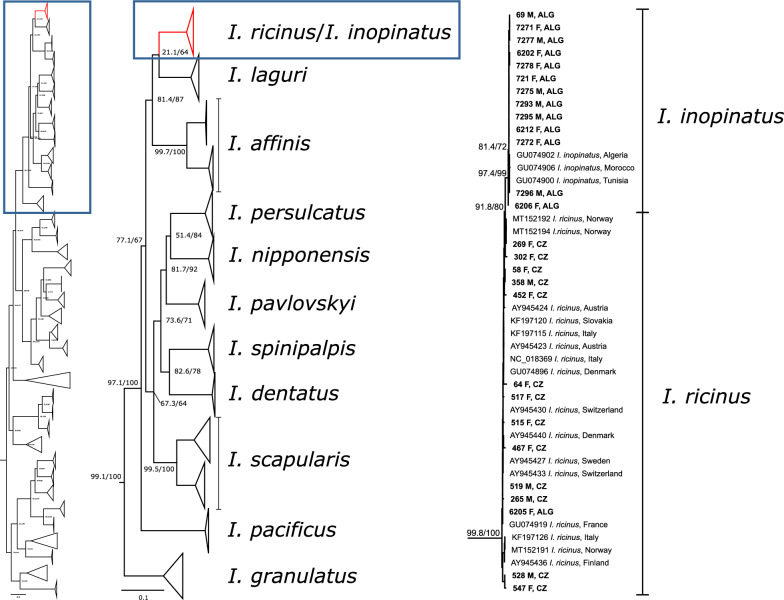
Phylogenetic tree of ticks based on the *COI* gene depicting a very close relationship between *I. ricinus* and *I. inopinatus*. Samples from this study are indicated in bold font. *CZ* Czech Republic, *ALG* Algeria

Amplification and sequencing of the *COI* gene were done on 285 ticks (245 CZ and 40 ALG). In the phylogenetic analysis of the *COI* gene, all representative sequences from this study form a single clade together with the *I. ricinus* and *I. inopinatus* sequences retrieved from the GenBank database (Fig. 4). Although the bootstrap support of the clade is low, its resolution from the most closely related *I. laguri* is clear. All *I. inopinatus* sequences (as assigned based on the *TROSPA* analyses or by their name in GenBank) form a separate, highly supported subclade, although with very short branch length. Other *Ixodes* spp. form well-distinguished and supported clades, some with the intraspecific subclade structure (e.g., *I. affinis* and *I. persulcatus*) (Additional file 1: Figure S4).

**Fig. 5:**
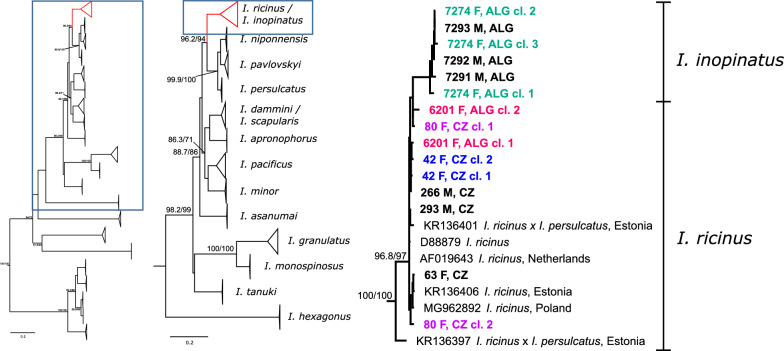
Phylogenetic tree of ticks based on the *ITS2* gene Samples from this study are indicated in bold font. *CZ* Czech Republic, *ALG* Algeria. Matching colors indicate sequences after cloning to resolve sequence ambiguities

In addition to the *TROSPA* gene, two other nuclear markers were also amplified and sequenced from the subset of our tick samples. After direct sequencing, the *ITS2* region yielded high-quality chromatograms only from 24 ticks (13 CZ and 11 ALG). In other samples, a sudden loss of the sequencing signal followed by an apparently mixed product chromatogram was observed. PCR products from 15 samples (eight CZ including two ticks assigned as ambiguous by multiplex PCR, and seven ALG) were cloned and 71 individual clones were sequenced (4–7 clones per sample). Among these, 37 unique clones (18 CZ and 19 ALG) were observed. Phylogenetic analyses of the available sequences representing various *Ixodes* spp. showed a pattern similar to that of the *COI* *gene*. All *I. ricinus* and *I. inopinatus* sequences (directly sequenced and cloned in this study as well as from GenBank) form a single, highly supported clade. All sequences of *I. inopinatus* (as assigned by the *TROSPA* gene) assembled into the unsupported subclade (Fig. 5, Additional file 1: Figure S5). Clones originating from a single individual always fell into a single subclade (*I. ricinus*/*inopinatus*). All clones from the two ambiguous samples (80F and 42F) clustered within the *I. ricinus* subclade.

A part of the *calreticulin* gene was amplified and sequenced from 34 ticks (19 CZ and 15 ALG). Sequences with 0–2 double peaks (ambiguous bases were assigned) from 26 ticks (15 CZ and 11 ALG) were used for the phylogenetic analyses. The fragment was not suitable for distinguishing the *Ixodes* species since no clades were formed in the phylogeny (Additional file 1: Figure S6).

## Discussion

Accurate identification of ticks at the species level is critical from several perspectives including distribution mapping, life cycle, and host range/preference, and most importantly for vector capacity for pathogens. Typically, methods based on the morphology and sequencing of 16S rDNA have been used for tick identification and description of new species [26, 28, 29], and other genes have rarely been used [27, 30, 33]. However, morphological identification relies on acarological expertise and specimen quality, and is rather time-consuming on large datasets. Furthermore, very low morphological variability makes it impossible to use for the identification of closely related taxa [9]. Sequencing of 16S rDNA is considered a gold standard for the identification of ticks and many other organisms, including bacteria. However, *I. ricinus* could not be differentiated from *I. inopinatus* by the commonly used 16S rDNA fragment [13, 16] due to the high haplotype diversity of this gene [8, 10, 11, 16, 22–25]. The *COI* gene is another common marker used for species delineation within the animal kingdom (e.g., the BOLD [Barcode of Life Data System] database); however, similarly to 16S rDNA, its analyses do not have the power to detect potential hybridization between closely related species. Mitochondrial markers are typically inherited uniparentally and therefore do not reflect the genetic history of an organism [43, 44].

Nuclear genes can reveal potential hybridization [35, 36], but these often have several copies resulting in mixed chromatograms in Sanger sequencing and the consequent need for cloning. So far, the *TROSPA* gene was the only marker that consistently distinguished tick populations from North Africa and Europe [5]. Internal transcribed spacers 1 and 2 (*ITS1* and *ITS2*) are useful for subtyping due to the high intraspecific diversity [5]; however, for many tick species, sequences for these loci are not available in GenBank [45]. *Calreticulin* was found to be completely inappropriate for distinguishing tick species, which is consistent with Babkin et al. [46].

Clearly, the *TROSPA* and *ITS2* genes seem to be good candidates for differentiation of the North African lineage of ticks referred to as *I. inopinatus* from the European population of *I. ricinus*. However, relatively small differences in these two markers between *I. ricinus* and *I. inopinatus* (in comparison to differences among other *Ixodes* species) opened a question of the natural gene flow between tick populations in North Africa and Europe. Our *TROSPA* data indicate natural hybridization followed by gene introgression and that hybrids of *I. ricinus* and *I. inopinatus* survive and may backcross the European parental population potentially resulting in unidirectional introgression [47]. However, this needs to be investigated further with larger sets of ticks, especially from North Africa.

Distribution patterns of arthropods, amphibians, reptiles, and mammals demonstrate biogeographical affinities between Europe and North Africa at the species level [48]. The distribution of primarily Palaearctic species across the Mediterranean has attracted considerable attention, showing North Africa as a refugium and differentiation center for Western Palaearctic thermophilic species. However, this applies to non-flying organisms only. *Ixodes ricinus* is a tick species commonly reported on birds (especially nymphs and larvae) [45], including migratory species [21, 49, 50]. We hypothesize, that *I. inopinatus* is adapted to climatic conditions in North Africa, and possibly the southernmost areas of Europe. African ticks are likely regularly carried by migratory birds between North Africa and Europe, as documented in the case of *Hyalomma* spp. [51, 52] as well as *I. ricinus* and *I. inopinatus* [53].

Since we did not find any signs of hybridization in North Africa, we hypothesize, that *I. ricinus* ticks from higher latitudes and their hybrids with the African population do not survive well in North African climate and that only North African ticks carried to Europe successfully hybridize and backcross with *I. ricinus* in Europe. However, it is important to point out that our data set from Algeria is much smaller than that from the Czech Republic, and follow-up studies are needed. To address this, we are currently conducting a surveillance study of ticks across Italy and additional sampling in Algeria (in preparation). The surveillance of ticks on migratory birds in the Czech Republic is also underway.

Similarly to the study from Germany [54], our data put into question studies reporting *I. inopinatus* from Central Europe based on morphology and/or sequencing of 16S rDNA, and we suggest that these should be re-examined. Even when *TROSPA* and other nuclear genes were used, double peaks and signs of hybridization and introgression have not been reported previously. In conclusion, we offer a fast and reliable multiplex PCR method for the identification of *I. ricinus* and *I. inopinatus*. Morphological similarity to *I. ricinus* and phylogenetic analyses both suggest African *I. inopinatus* to be “a species in statu nascendi” evolving from *I. ricinus*. Additional studies on the genetic diversity and the full genome sequencing of *Ixodes ricinus*/*inopinatus* in North Africa and regions of likely sympatry with *I. ricinus* in Europe (Spain, Portugal, Italy) are needed. Questions including the potential differences in vector competence between *I. ricinus* and *I. inopinatus* and their hybrids remain to be answered.

### Supplementary Information

**Additional file 1: Figure S1.** Multiplex PCR.** Figure S2.** *TROSPA* gene phylogeny.** Figure S3.** 16S rRNA phylogeny.** Figure S4.** *COI* gene phylogeny.** Figure S5.** *ITS2* phylogeny.** Figure S6.** *Calreticulin* gene phylogeny.

**Additional file 2: Table S1.** List of 23 SNPs in the *TROSPA* intron.** Table S2.** Sequences used for phylogenetic analysis.

## Data Availability

The representative nucleotide sequences generated in the present study and used in the phylogenies have been deposited in GenBank (https://www.ncbi.nlm.nih.gov/) under the accession numbers OQ981335-63 for 16S rRNA, OQ981450-76 for the *COI* gene, OQ991204-18 for *ITS2*, OQ999531-56 for the *calreticulin* gene, and OQ999557-628 for the *TROSPA* *gene*. The datasets used and/or analyzed during the current study are available from the corresponding author upon a reasonable request.
