# Supplementary material for: Genetic analysis challenges the presence of Ixodes inopinatus in Central Europe: development of a multiplex PCR to distinguish I. inopinatus from I. ricinus
Source: Parasit Vectors. 2023 Oct 9;16:354. doi: 10.1186/s13071-023-05971-2 (PMC10561450; doi:10.1186/s13071-023-05971-2)

Figure S1

Multiplex PCR on TROSPA gene

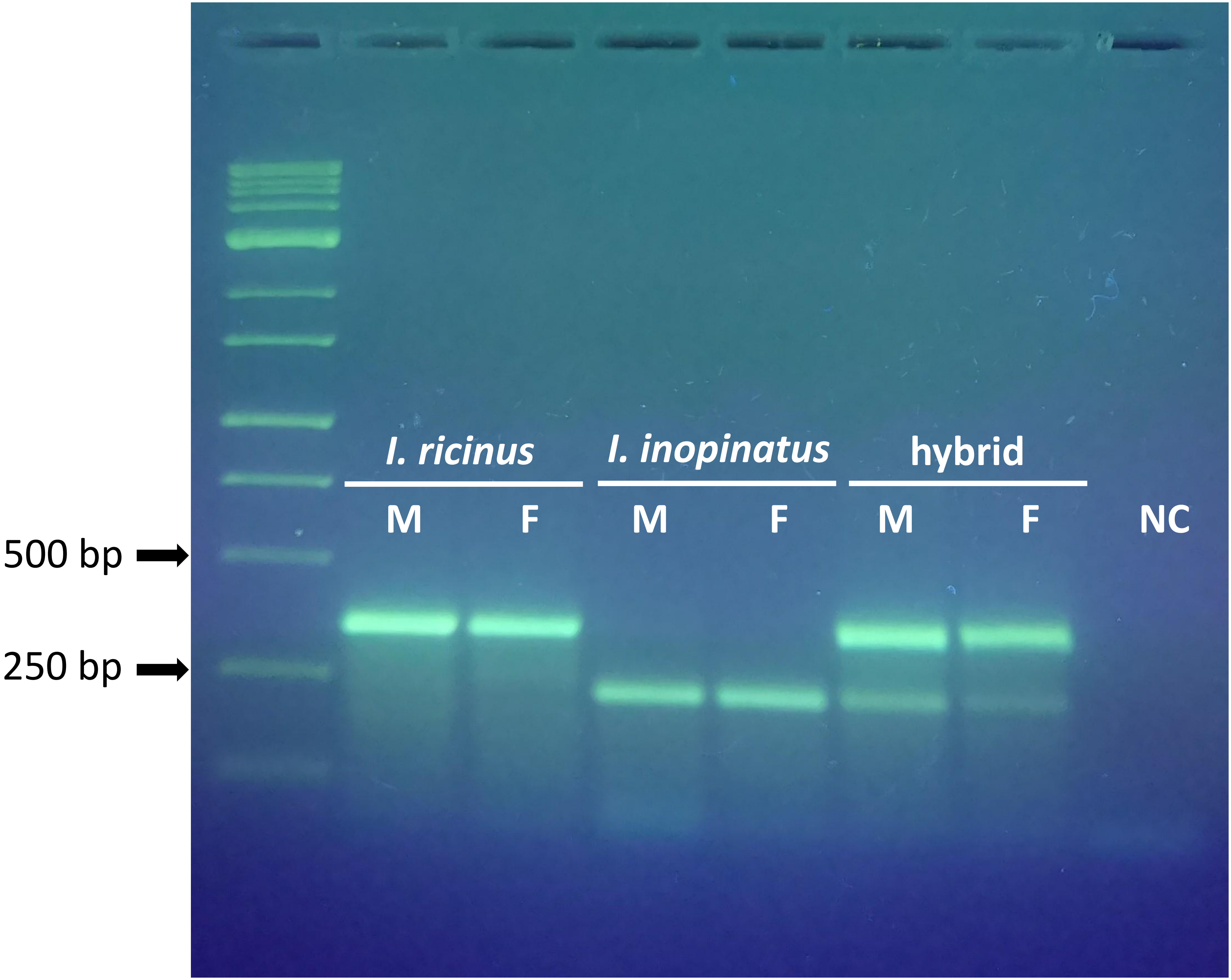

Figure S2
TROSPA gene

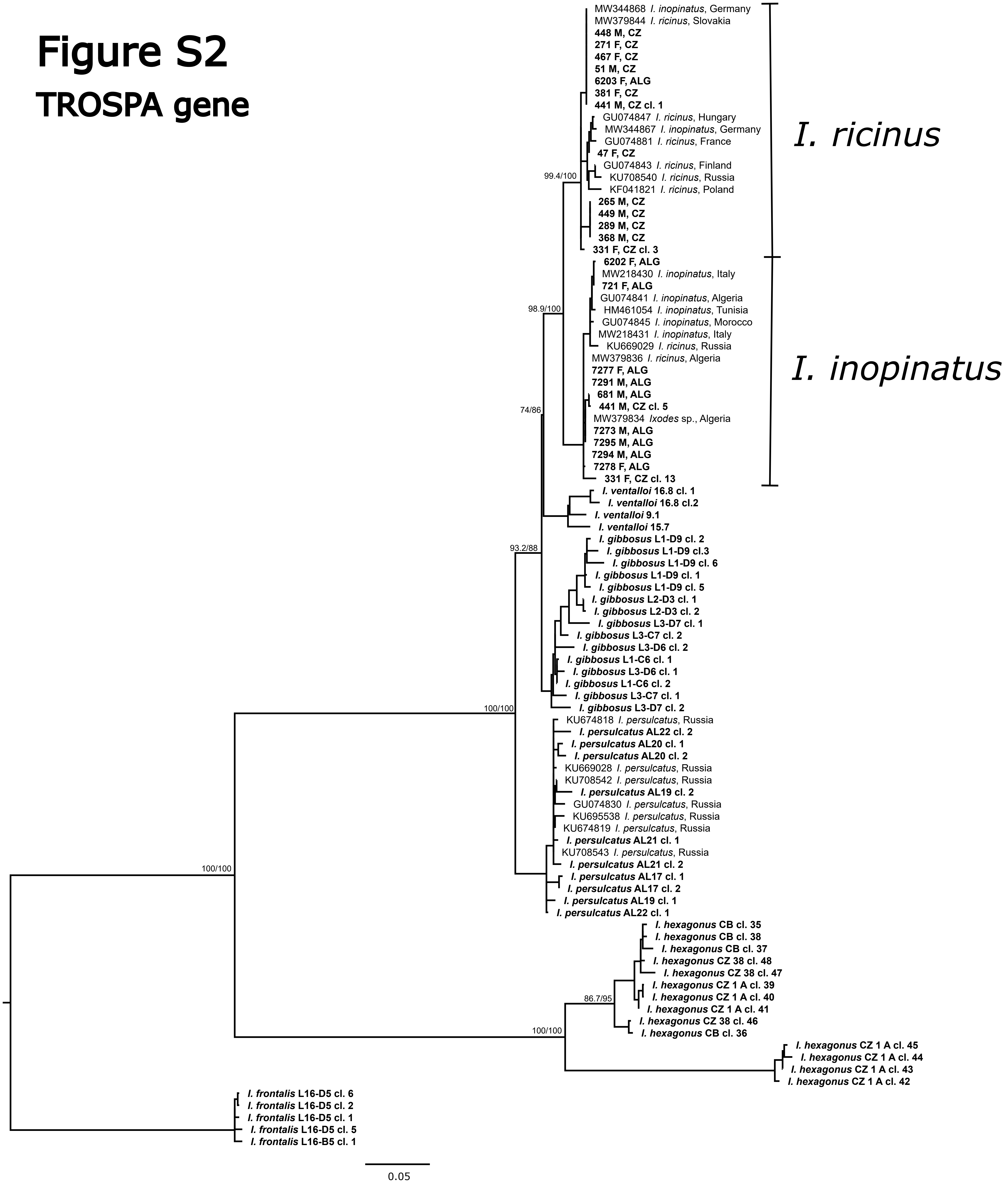

Figure S3

16S rRNA gene

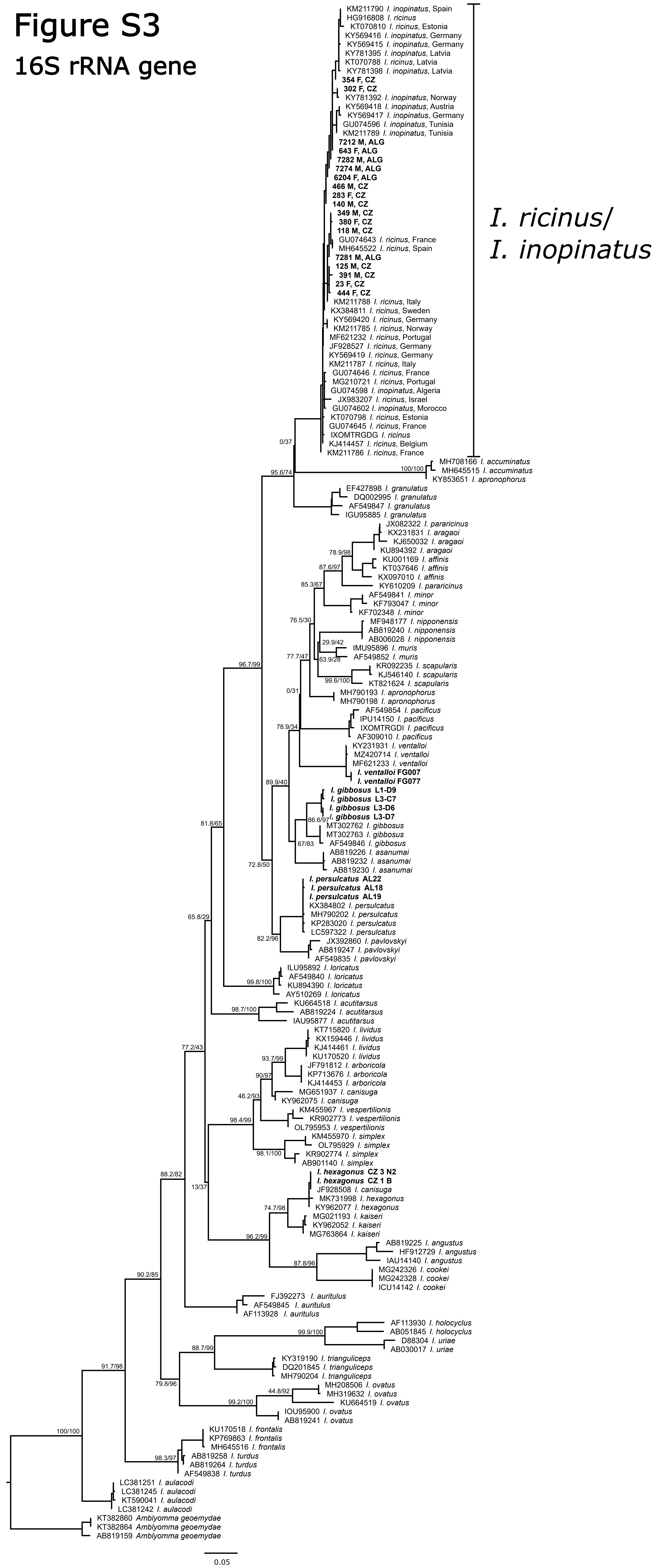

Figure S4

COI gene

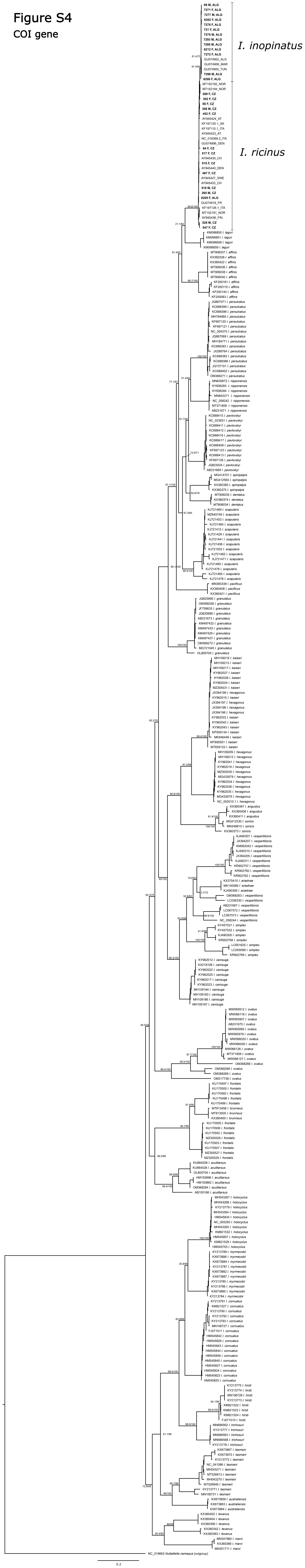

Figure S5
ITS2

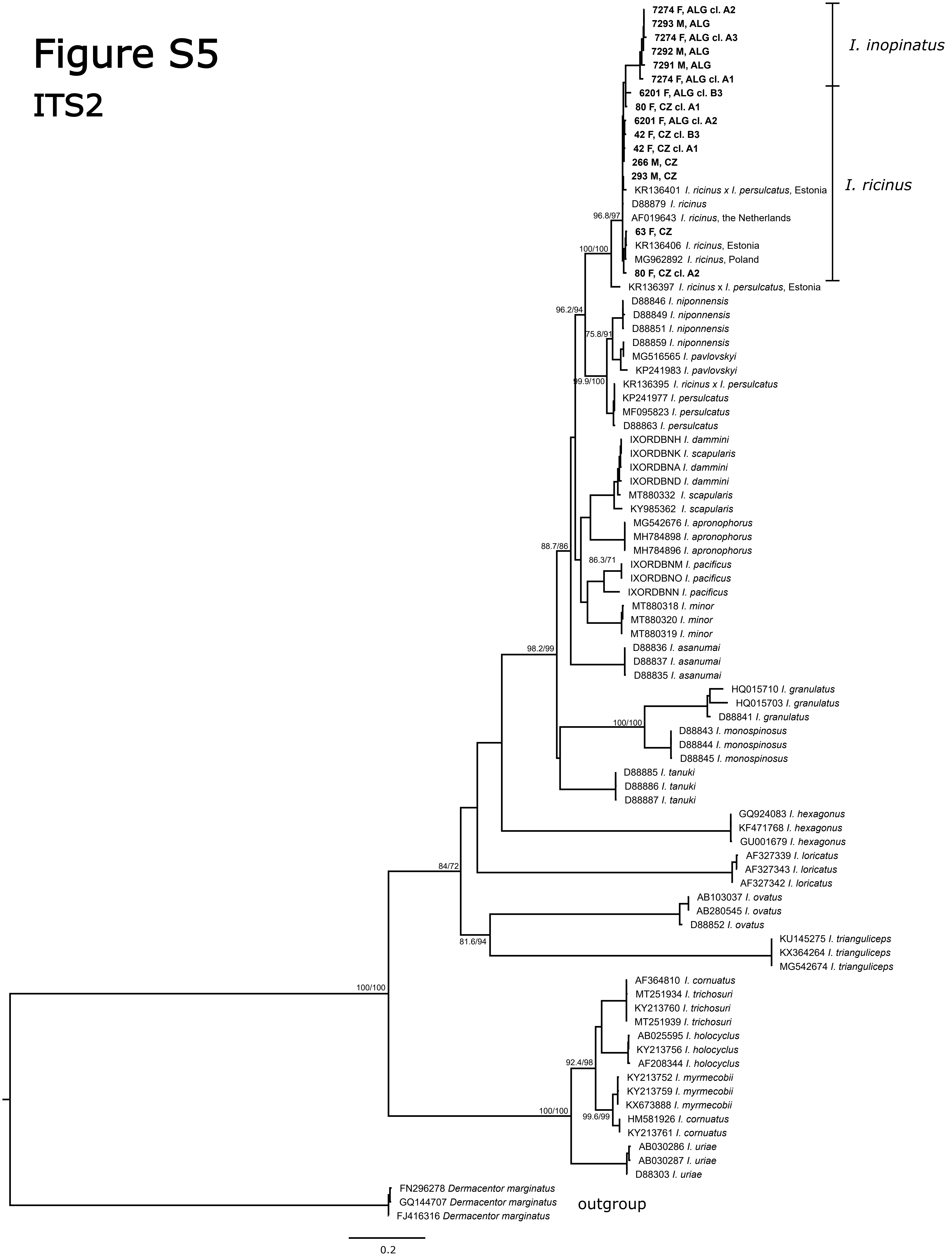

Figure S6

Calreticulin gene

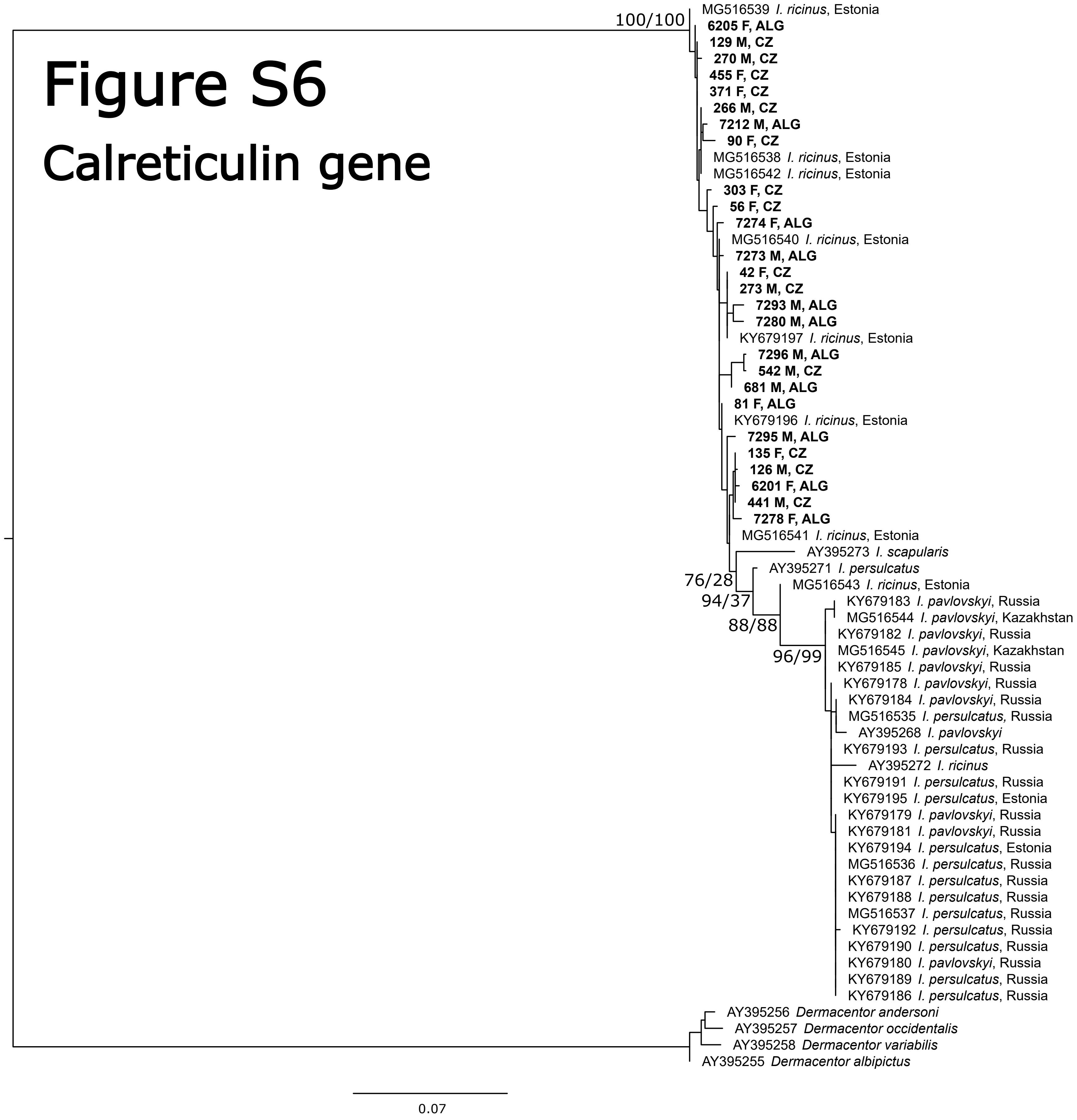

Supplement: Supplementary file 1 — Additional file 1: Figure S1. Multiplex PCR. Figure S2. TROSPA gene phylogeny. Figure S3. 16S rRNA phylogeny. Figure S4. COI gene phylogeny. Figure S5. ITS2 phylogeny. Figure S6. Calreticulin gene phylogeny. [file 13071_2023_5971_MOESM1_ESM.pdf]
