# Supplementary material for: Genetic analysis challenges the presence of Ixodes inopinatus in Central Europe: development of a multiplex PCR to distinguish I. inopinatus from I. ricinus
Source: Parasit Vectors. 2023 Oct 9;16:354. doi: 10.1186/s13071-023-05971-2 (PMC10561450; doi:10.1186/s13071-023-05971-2)
Supplement: Supplementary file 2 — Additional file 2: Table S1. List of 23 SNPs in the TROSPA intron. Table S2. Sequences used for phylogenetic analysis. [file 13071_2023_5971_MOESM2_ESM.pdf]

Table S1. List of 23 SNPs in the TROSPA intron differentiating *I. ricinus* and *I. inopinatus*. Sequence KF041821 is used as a reference for SNP positions.

**Polymorphism**	**KF041821**	***I. ricinus***	***I. inopinatus***
S	463	G	C
R	494	A	G
S	514	G	C
R	519	A	G
K	520	T	G
K	522	G	T
R	548	A	G
R	563	G	A
R	595	G	A
Y	607	C	T
R	646	G	A
Y	648	C	T
M	742	A	C
Y	782	T	C
K	810	T	G
Y	811	T	C
R	843	G	A
R	844	G	A
Y	849	T	C
Y	853	C	T
W	857	A	T
Y	862	T	C
Y	864	C	T

Table S2. Sequences used for phylogenetic analysis

Gene	No. of sequences from this study	No. of sequences from GenBank	Alignment algorithm	Total length of the alignment (columns)	Evolution model
16S rRNA	29	144	ClustalW	462	GTR+F+R4
TROSPA	72	23	Mafft	956	TPM2+F+I
COI	27	287	ClustalW	1556	GTR+F+I+G4
ITS2	15	52	ClustalW	851	HKY+F+R2
Calreticulin	26	39	ClustalW	1140	TN+F+R2
